# Complete Genome Sequence of *Kluyvera* sp. CRP, a Cellulolytic Strain Isolated from Red Panda Feces (Ailurus fulgens)

**DOI:** 10.1128/mra.00067-22

**Published:** 2022-03-28

**Authors:** A. C. H. Wai, G. K. K. Lai, S. D. J. Griffin, F. C. C. Leung

**Affiliations:** a Shuyuan Molecular Biology Laboratory, The Independent Schools Foundation Academy, Hong Kong, SAR, China; Indiana University, Bloomington

## Abstract

The enterobacterium genus *Kluyvera* is widely distributed in the environment and a rare source of infection in humans. *Kluyvera* sp. strain CRP was isolated from feces of a healthy, captive Chinese red panda (Ailurus fulgens), and its complete genome (5,157,963 bp, 54.80% GC content) was established through hybrid assembly.

## ANNOUNCEMENT

*Kluyvera* species are coliforms that are widely distributed in the environment and have been isolated from freshwater (1), seawater (2), sewage (3), and soil (4, 5); from synanthropic spiders (6) and flies (7); from cows (8), Egyptian fruit-bats (9), and sea turtles (10); and from the rhizosphere (11) and as endophytes (12), where they may promote plant growth (13, 14) and protect against disease (15). In humans, *Kluyvera* infections are uncommon but are often persistent and even fatal (16–19).

*Kluyvera* sp. strain CRP was isolated from the feces of a healthy Chinese red panda (Ailurus fulgens) housed at Ocean Park, Hong Kong. Previous characterization of gut microbiota in this species has indicated the presence of extensive carbohydrate metabolism, including cellulose-degrading pathways consistent with a mainly bamboo diet (20).

First, 1 g of fresh feces was vortexed in 9 mL of 0.9% (wt/vol) NaCl and briefly allowed to settle before serial dilution. Then, 100 μL of the 1,000× extract was spread onto carboxymethylcellulose (CMC) agar (21) and incubated at 27°C for 48 h. Colonies displaying strong growth were picked and passaged to purity on Luria agar. Cellulolytic activity was confirmed by incubation on CMC agar followed by staining with Gram’s iodine (22). Isolate CRP, which showed the greatest activity, was passaged on Luria agar 9 times before a single colony was incubated in Luria broth for 24 h prior to DNA extraction (Invitrogen; PureLink genomic DNA minikit).

Paired-end short-read sequencing libraries were prepared using the Nextera XT DNA library preparation kit and sequenced via the Illumina MiSeq platform using v3 chemistry (2 × 300 bp). Adapter sequences were removed using Trimmomatic v0.32 (23), and reads were quality-filtered and trimmed, producing 1,204,982 read pairs (mean length, 297 bp) totaling ∼358 Mbp. Long-read libraries, prepared from the same extracted DNA using the rapid barcoding kit SQK-RBK004, were sequenced via Oxford Nanopore’s SpotON flow cell vR9, a MinION sequencer, and MinKNOW v3.1.8 software, with base-calling using Guppy v2.1.3. The final long-read data set, trimmed using Porechop v0.2.4 (24, 25), totaled 40,127 reads (494 Mbp) with a median length of 15,833 bp (*N*_50_, 60,789 bp). Default parameters were used for all software unless otherwise specified.

The complete genome sequence combined Illumina and MinION datasets using Unicycler v0.4.3 (26), yielding a circular chromosome of 5,157,963 bp, which was submitted to NCBI PGAP v5.0 (27) and to PATRIC (28) for annotation.

Mash/MinHash using PATRIC (29) found CRP to be closest to *Kluyvera* genomospecies 3 strain PO2S7 (GenBank accession number CP050321), with an average nucleotide identity of 98.83% (30, 31). A total of 10 copies of 6-phospho-beta-glucosidase (EC 3.2.1.86) are present, together with multiple copies of cellobiose phosphotransferase (EC 2.7.1.205) and the *bcs* operon (32). In susceptibility testing (Liofilchem), CRP showed resistance to ampicillin (10 μg) consistent with the presence of a CTX-M-40 beta-lactamase (33, 34) identified using CARD (35).

### Data availability.

The complete genome sequence and raw sequence data for *Kluyvera* sp. CRP are available through NCBI under BioProject number PRJNA758164 and GenBank accession number CP082841. The genome sequence for *Kluyvera* genomospecies 3 strain PO2S7 can be found under GenBank accession number CP050321.
